# Power in global governance: an expanded typology from global health

**DOI:** 10.1186/s12992-019-0515-5

**Published:** 2019-11-28

**Authors:** Suerie Moon

**Affiliations:** Global Health Centre, Graduate Institute of International and Development Studies, Geneva, Switzerland

**Keywords:** Power, Typology, Global governance, Global health, Political determinants, Complex adaptive system, Power asymmetry, Power disparity

## Abstract

The exercise of power permeates global governance processes, making power a critical concept for understanding, explaining, and influencing the intersection of global governance and health. This article briefly presents and discusses three well-established conceptualizations of power—Dahl’s, Bourdieu’s, and Barnett and Duvall’s—from different disciplines, finding that each is important for understanding global governance but none is sufficient. The conceptualization of power itself needs to be expanded to include the multiple ways in which one actor can influence the thinking or actions of others. I further argue that global governance processes exhibit features of complex adaptive systems, the analysis of which requires taking into account multiple types of power. Building on established frameworks, the article then offers an expanded typology of eight kinds of power: physical, economic, structural, institutional, moral, discursive, expert, and network. The typology is derived from and illustrated by examples from global health, but may be applicable to global governance more broadly. Finally, one seemingly contradictory – and cautiously optimistic – conclusion emerges from this typology: multiple types of power can mutually reinforce tremendous power disparities in global health; but at the same time, such disparities are not necessarily absolute or immutable. Further research on the complex interaction of multiple types of power is needed for a better understanding of global governance and health.

## Background

The past decade has witnessed increasing scholarly attention to the role played by power in shaping global governance, and the implications for global health [1]. A key conclusion of the WHO Commission on the Social Determinants of Health was that protecting health required “tackling the inequitable distribution of power, money and resources [2].” In 2014, the Lancet Commission on Global Governance for Health published its final report focusing on transnational political determinants of health, and used the word “power” 83 times [3, 4]. The same year, political scientist Jeremy Shiffman called attention to the “critical need to investigate how epistemic and normative power get exercised in the global health field,” prompting an outpouring of reactions from other scholars [5]. Theories of power, concluded Radhika Gore and Richard Parker in a 2019 special issue of *Global Public Health* dedicated to power, had not adequately informed global health scholarship [6]. And, assessing the literature on power in health systems in low- and middle-income countries (LMICs), Veena Sriram and colleagues concluded that further methodological development was needed to understand how power affects health [1].

A perennial concern is not merely the existence of power, but the disparities or imbalances of power that result in poorer health for the less powerful. Frequently noted power asymmetries in global governance include those between the Global North and South, between richer and poorer populations or organizations, between males and females, between health and economic or national security interests, and between business, states, and civil society. However, these generalizations—while providing useful mental shortcuts—can also obscure the more nuanced power dynamics that characterize social interactions. Importantly, grouping actors into a binary classification of “powerful” or “powerless” fails to recognize the many ways in which actors—even those traditionally characterized as “weak” in International Relations (IR) studies—are able to exert influence in global governance [6]. In her response to Shiffman, Kelley Lee pointed out not only that “global health is shot through with power relationships” but also that “it takes many forms [7].” What are those forms? And what does understanding these forms offer for scholarship and practice?

In this article, I provide a brief overview of how the concept of power in global governance has evolved in scholarship, followed by a typology of eight kinds of power derived from the field of global health. This typology grew out of global health, but may be applicable to other arenas of global governance as well. Therefore I refer throughout this article to “global governance processes affecting health,” which may take place within the health sector (sometimes referred to as “global health governance”) and outside of it (sometimes referred to as “global governance for health,” although the terminology is not consistently applied in the literature) [8, 9].

### How have scholars conceptualized power in global governance?

The conceptualization of power in the social sciences has evolved to produce a more nuanced understanding of how power operates in societies. A brief examination of several influential theorists from different disciplines, selected for relevance to this discussion, highlights the multiple types of power generally recognized as relevant. An in-depth review of power is beyond the scope of this article: I merely highlight several key ideational shifts that have shaped how power in global governance is understood today.

#### Dahl: power as coercion

The US political scientist Robert Dahl famously conceptualized power as: “A has power over B to the extent that he can get B to do something that B would not otherwise do [10].” The element of coercion in Dahl’s definition is central to many notions of power in IR studies, particularly in the realist school [11]. Translated into IR theory, Dahl’s definition became “the ability of states to use material resources to get others to do what they otherwise would not do” [11]. But what if the counterfactual—what B would “otherwise do”—is not clear, or not known? What if A is able to persuade B overtly, such that B wants to do what A wants? What if A influences B covertly or unintentionally, such that B (perhaps also A) is unaware that A is influencing what B decides to do? What if a decision by A at one time influences what B does many years later? As sociologists have long argued, power can operate in far more subtle and indirect ways than through coercion—or even persuasion—alone [12–15].

#### Bourdieu: power as capital

Key ideas from the renowned social theorist Pierre Bourdieu have received growing attention from IR scholars [16, 17]. Political theorist Stefano Guzzini characterized Bourdieu’s approach to power as combining the insights of Steven Lukes and Michel Foucault [18]. Lukes had argued that power exists and matters not only when it is overtly exercised, as in situations of contested political decision-making, but also when it operates in less obvious ways, such as non-decision-making and agenda-setting [14]. Taking this argument even further, Foucault argued that “power is everywhere, not because it embraces everything but because it comes from everywhere” [13], highlighting the ways in which multiple actors may wield power in any social interaction. Both Lukes and Foucault held that conceptualizing power narrowly as overt domination ignored a large part of the picture.

An important advance made by Bourdieu was to conceptualize power as capital: the two concepts “amount to the same thing” [12]. Hanefeld and Walt highlighted the utility of Bourdieu’s four forms of capital—economic, cultural, social, and symbolic—for understanding power in global health [19]. They argued that actors with economic capital, such as the Bill and Melinda Gates Foundation (BMGF), could translate it into the cultural capital that derives from “education, academic titles, epistemic knowledge and recognised experience” by funding academic research, for example [19]. Cultural capital could be further amplified through access to social capital, “the links and connections between networks of organisations and individuals,” as when networks elevate the attention paid to certain research results or policy proposals over others [19]. Finally, Bourdieu defined symbolic capital as “the acquisition of a reputation for competence and an image of respectability and honourability [20].” WHO guidelines, argued Hanefeld and Walt, carried symbolic capital that often resulted in countries changing their national policies in response, although the WHO has no authority to mandate such changes [19]. By conceptualizing power as capital, Bourdieu emphasized its fungibility and productiveness—the ways in which one kind of power could be transformed into and amplify others.

Bourdieu used these categories of capital to explain the construction and persistence of social structures in middle-class France in the 1970s–80s. His concepts translate readily to global health (among many other arenas of social interaction), but are inadequate for explaining processes of global governance. For example, the actual or threatened use of physical force has dominated scholarly analysis of international politics, but is absent from Bourdieu’s typology. Similarly, his typology does not account for the power that states derive from the state-based structure of the international system (structural), or the power vested in the rules and procedures of formalized international decision-making (institutional)—both of which have been central considerations in IR scholarship [21, 22]. A typology of power tailor-made for global governance could offer additional insights to Bourdieu.

#### Barnett and Duvall: power in global governance

Arguably the most influential effort to provide such a typology has been Barnett and Duvall’s 2005 article, “Power in international politics” [11], and their edited volume published the same year, *Power in Global Governance* [23]. Like Bourdieu, Barnett and Duvall also argue for a more nuanced, “polymorphous” conceptualization of power in the analysis of international politics. Rather than the coercive realist concept of one state getting another state to do what “it does not want to do,” Barnett and Duvall define power more broadly as “the production, in and through social relations, of effects that shape the capacities of actors to determine their circumstances and fate [11].” Their taxonomy of four types of power—compulsory, institutional, structural, and productive—seeks to encompass both “power over” and “power to,” and to address the perennial debates over the relative roles of agency vs structure. I review their typology in some detail here, as my own typology adapts and builds directly on theirs.

Barnett and Duvall define compulsory power as “direct control over another,” and argue that it is best understood through the eyes of the object (B, above) rather than the wielder of power (A, above). In this category they include material (e.g. military, economic), symbolic, and normative power, and argue that states, NGOs, international organizations, militias, multinational firms, and many other types of actors may all wield it [11]. A drawback with this definition of compulsory power, however, is that in focusing on the effects and objects of power it de-emphasizes the actors who wield power and how they do so. Further, the actual exercise of truly compulsory power—in which the object of such power, B, has no choice but to do what A wants—is relatively rare. In most situations, actors retain at least some degree of agency; even when direct physical force is exercised, they can choose to resist or submit, and only in the most extreme circumstances are they truly robbed of all choice. B may choose to do what A wants in order to avoid negative consequences, such as being physically harmed, impoverished, or socially stigmatized, for example—but, alternately, B may accept those consequences. I argue that, in the typology offered below, separating out the different kinds of power that Barnett and Duvall aggregate under “compulsory” can provide important additional analytical traction.

Barnett and Duvall’s second category, institutional power, is defined as “actors’ control over socially distant others” with a focus “on the formal and informal institutions that mediate between A and B, as A, working through the rules and procedures that define those institutions, guides, steers, and constrains the actions (or nonactions) and conditions of existence of others [11].” A central distinction that Barnett and Duvall emphasize is that compulsory power involves direct action by A on B, whereas institutional power involves A exerting power indirectly on B (or many Bs) through the diffuse channels of institutional arrangements. In contrast to their conceptualization of compulsory power, which focuses on the effects of diverse types of power, their definition of institutional power focuses on the source and channels by which a type of power is exercised. However, the distinction between direct and indirect exertion of power is not always clear. Material, symbolic and normative power (which the authors include under compulsory power) do not always act directly on the object, but can also operate indirectly—through the rules and procedures that constitute institutions, or in an even less structured manner through normative claims of advocates (for normative power) or of an actor with high social status (for symbolic power). I argue for narrowing down the definition of institutional power to focus on rules and decision-making procedures. Because institutions reflect the distribution of power when they were created, but may persist long afterwards, they can also perpetuate power disparities between those who are advantaged and disadvantaged by a particular set of rules and procedures [3, 11].

Barnett and Duvall’s third category is structural power, defined as “the co-constitutive, internal relations of structural positions that define what kinds of social beings actors are….that is, a direct constitutive relation such that the structural position, A, exists only by virtue of its relation to structural position, B [11].” Further: “the social relational capacities, subjectivities, and interests of actors are directly shaped by the social positions that they occupy,” and such structures are likely to privilege some over others [11]. Highly relevant for understanding global governance is the structure of the Westphalian international system, based on the state as the principal authority over its population and territory. Particularly relevant for global health are theories that pay attention to the position of developing countries in the global structure, such as world systems theory (which divides states into core, periphery and semi-periphery) [24]. Like their conceptualization of institutional power, this category focuses on the source of power and not its effects. However, a key challenge is distinguishing between institutional and structural power: both emphasize the social relations between A and B. For example, the power a physician has over a patient can be considered both structural—they directly co-constitute each other’s position in a given setting in society (e.g. a hospital)—or institutional, as when physicians constrain what patients can do directly or indirectly (e.g. through rules for prescribing medicines, or norms governing the functioning of complex healthcare facilities and systems). Clearer distinctions are needed.

Barnett and Duvall’s fourth category is productive power, defined as “the constitution of all social subjects with various social powers through systems of knowledge and discursive practices of broad and general social scope.” They contrast structural power, which emphasizes relations of dominance and subordination between two actors, with productive power, which refers to more “diffuse and contingent social processes produc[ing] particular kinds of subjects, fix meanings and categories, and creat[ing] what is taken for granted and the ordinary of world politics [11].” This broad conceptualization of productive power usefully highlights that social processes of categorization, framing, and knowledge-production do constrain and shape thinking, beliefs, and action. However, this definition does not identify who wields this kind of power (to a greater or lesser extent) or how it might be intentionally exercised—actors are ubiquitous, and agency is absent in this picture. The definition is also very broad. Returning to the physician–patient example, classifying a person as a patient can be seen as a result of both productive power (since the patient is a “particular kind of subject”) and structural power (since the patient can be considered to be co-constituted in relation to the physician, often in a relationship of dominance).

Barnett and Duvall’s argument for a broader typology of power was an important challenge and major contribution to IR, which had long privileged a unidimensional realist conceptualization of power as coercion. However, they ultimately adopted a limited conceptualization of power—“restricted to the production of particular kinds of effects, namely those on the capacities of actors to determine the conditions of their existence [11].” They did not go so far as to conceptualize power as “the production of any and all effects and thus as nearly synonymous with causality” [11], explicitly excluding persuasion and collective decision-making processes (but including the social structures and constitution of social subjects that facilitate persuasion, collective-decision-making, and many other pathways of influence). Thereby, their typology does not cover the full arsenal of power that actors wield in global governance. Their typology also usefully emphasizes the distinction between direct and indirect power, but simultaneously combines the effects, sources, and channels through which power is exercised. The typology presented in Part 4 below, focused on the source of an actor’s power, adapts and seeks to build on the foundation they constructed.

#### Sriram et al.: power in LMIC health policy and systems

In addition to theorization on power from the broader social sciences, the field of global health has also taken a growing interest in the concept. Veena Sriram and colleagues conducted a thorough review of the state of the literature on power as it relates to health policy and systems of LMICs, and generally found it “lacking” and a “neglected area of work [1].” Discussing various conceptualizations of power, they identified six sources of power that arise in the broader social science literature: technical expertise, political power, bureaucratic power, financial power, networks and access, and personal attributes [1]. While some of these categories overlap conceptually with Dahl, Bourdieu, and Barnett and Duvall, their review also usefully highlights elements that these other scholars neglected, such as the power linked to networks, and personal attributes such as charisma. Sriram et al. mention global governance, but focus on health policy and systems in LMICs. This focus yields insights particularly relevant for health, such as the emphasis on technical expertise or bureaucratic power, but ignores factors central to IR studies, such as physical force or institutional power.

Here I have highlighted the diversity of conceptualizations of power, and how understandings have shifted considerably over time. As no single conceptual framework suits all purposes, I hold that analyzing how power in global governance affects health would benefit from a tailor-made framework.

### Why is an alternate typology needed? Global health as a complex adaptive system and its implications for power analysis

Given the profound complexity of global governance processes [25], a broader and fuller conceptualization of power is needed. Peter Hill argued for understanding global health as a complex adaptive system in which many relatively autonomous actors continuously interact and adapt to each other, producing a system of a different character than the mere sum of its constituent parts [26]. Contemporary global governance processes are indeed characterized by hundreds, even thousands, of state and non-state actors simultaneously interacting and pursuing their objectives across multiple sectors, countries, times, and scales—sub-national, national, regional and global. The complex adaptive system they constitute can be characterized as tightly linked, governed by feedback loops, non-linear, self-organizing, and path-dependent [27]. This complexity implies that it is not adequate to conceptualize power simply as A’s effect on B (whether the exercise of power is direct or diffuse): we must rather take into account the simultaneous and reciprocal effects of actors A through Z on each other.

In addition, complex adaptive systems are often characterized by “butterfly effects” in which a small action in one part of the system can have significant unintended and/or unforeseen effects in other parts of the system. The butterfly effect implies that different types of power and actors traditionally characterized as weak in international affairs may have significant impacts on the system’s outcomes. Nor is it adequate to examine only a few ways in which actors exert power in the system—the tools of influence of a wealthy state differ from those of a poorer one, those of a multinational corporation differ from those of an international NGO, those of an expert differ from those of a journalist, and those of a religious leader differ from those of a grassroots social movement. While some scholars have argued for a more limited conceptualization of power to facilitate analysis [11, 18], I hold that, in a complex adaptive system, a full understanding of global governance requires taking into account all the ways in which actors can produce effects.

### What kind of power is exercised in global governance processes affecting health?

The following typology draws on the broader literature and builds on Barnett and Duvall, while seeking to address some of the critiques noted above. The typology has been informed by the literature on global governance/international relations and health, discussions with colleagues in academia and practice, participant and non-participant observation in global governance processes, and my own experience working with graduate students to analyze power in teaching and research over the past decade. Encompassing eight types of power, it aims to be comprehensive and broad rather than parsimonious (see Table 1). It emphasizes the different forms of power often available to various actor types. By focusing on actors, I hope to make the conceptual framework relevant for practitioners, whose strategy may be informed by a clear understanding of how other actors in their issue-area exercise power.

**Table 1:** Types of power in global governance, with examples from health

Type of power	Examples of actors wielding such power	Health-related examples of uses of such power
Physical	Militaries, militia, mercenaries, peacekeeping forces, police	Cordon sanitaire, quarantine
Economic	Wealthy governments, firms, foundations, individuals	Shaping WHO priorities through funding
Structural	Governments, traditional leaders	Governments levying taxes on tobacco sales
Institutional	Depends on institution: often governments, increasingly also firms and NGOs	Civil society delegation to Global Fund board voting on grantmaking policies
Moral	Religious leaders, social movement leaders, moral authorities	Speech by Nelson Mandela on de-stigmatizing HIV
Expertise	Academics, scientists, lawyers	Evidence on link between alcohol and cancer leading to changes in alcohol regulation
Discursive	Media, politicians, activists, public intellectuals	Contraception as sexual and reproductive right
Network	Any well-networked individual or group of individuals	Garnering invitations to prestigious committees or conference speaking roles

I define power in global governance as the ability to shape the thinking and/or actions of other actors in the global public domain. This definition takes an expansive view of power as largely synonymous with influence—that is, the ability to influence another actor *is* power. As argued above, limiting the concept of power to only “significant” instances of influence would risk missing vast parts of the picture, because seemingly minor acts of influence in one part of a complex adaptive system can have major effects elsewhere. The eight types of power identified here are particularly relevant in the field of health, but may apply to other arenas of global governance as well.

- **Physical**: Physical power is wielded when an actor uses or threatens to use physical force to shape the thinking or actions of other actors. At its extreme—when used to kill or severely disable—physical force can be truly compulsory: it can deny agency or choice to the object of force. But the mere threat of force may also wield enormous power. An example is when authorities detain individuals suspected of carrying infectious diseases, against their will, at airports and other ports of entry, to manage an outbreak. This type of power is generally available to governments (recalling Weber’s influential definition of the state as a “monopoly on the legitimate use of physical force” [15]). It may also be wielded by private actors such as militias, gangs, mercenary forces, or armed rebel groups.
- **Economic**: Economic power is wielded through the use of material resources (e.g. money, goods) to shape the thinking and actions of other actors. Any actor with access to material resources, such as governments, companies, foundations, or individuals, has access to such power. For example, an industry’s financial contribution to the election campaign of a legislator may shape the thinking and voting of that legislator to favor the industry’s interests.
- **Structural**: Structural power is wielded through the use of an actor’s position in the structures of society to shape the thinking and/or actions of other actors. The structures may be formal and legally recognized, such as the state, or traditional, such as castes or class. Governments, for example, have the structural power to regulate the behavior of private actors in their territories due to the state-based nature of the international system. A large multinational firm may have greater economic power than the low-income country in which it wishes to do business, but the structural power of the government means that the firm must still acquire the necessary permissions to do so from that country’s authorities.
- **Institutional**: Institutional power is wielded through an actor’s use of rules and decision-making procedures to shape thinking and action. For example, the decision-making rule in many intergovernmental organizations, including UN agencies such as the World Health Organization, is that each state gets one vote. This rule gives small states more power than they would wield in an institution where votes are proportional to a state’s contributions to the budget, such as the World Bank. Which actors benefit from institutional power depends on the specific institution at hand. The governing rules of the Global Fund and Gavi Alliance confer institutional power to both states and non-state actors as voting Board members, whereas the WHO’s Health Assembly privileges states. The line between social structures and social institutions may be blurred, but the key conceptual distinction I wish to make between the two is their relative degrees of durability: structure refers to the more deeply-embedded ways in which a society is organized, whereas institutions refer to the rules that actors create or agree upon in order to achieve a specific purpose (e.g. how to allocate development funding to candidate recipient countries).
- **Moral**: Moral power is wielded when an actor shapes the principles that others believe to be right or wrong, and the actions that may then follow. For example, widely respected leaders such as Nelson Mandela or Archbishop Desmond Tutu could shape the thinking of millions, including political leaders, regarding what was ethical behavior towards people living with HIV. Actors with this type of power, such as religious, civil society, political or philosophical leaders, are deemed to have moral authority by at least some elements of society. Moral power may also be wielded “from below,” as when grassroots activists build social movements that change broader norms regarding what is acceptable in society [28]. This is similar to Bourdieu’s concept of symbolic capital, but with a more explicit focus on values.
- **Expert**: Expert power is wielded when an actor shapes what others consider to be legitimate knowledge, and therefore what they understand to be factually true or correct [29, 30]. For example, experts who produce research on causal relationships shape influential WHO guidelines on a wide range of subjects, from alcohol to meat consumption, from the classification of disease to approaches for treating them. The Global Burden of Disease project and the Institute for Health Metrics and Evaluation have profoundly shaped estimates of disease incidence, prevalence, and burden [31]. Such power is available to those recognized to be experts, including but not limited to academics or think tank-researchers, and is particularly important in the health sector where expert knowledge is accorded great weight. It is analogous to (albeit narrower than) Bourdieu’s cultural capital, derived from academic credentials or other markers of knowledge, or Haas and Foucault’s technical expertise.
- **Discursive**: Discursive power is wielded when actors shape the language others use to conceptualize, frame, and thereby define and understand an issue. For example, different actors can influence whether contraception is referred to as “birth control,”” family planning,” “maternal and child health,” or “sexual and reproductive health.” The language used has implications for the subsequent availability and uptake of contraception. Discursive power is available to the wide range of actors engaged in a public debate, including actors traditionally considered to be powerful such as government officials, and those less so, such as civil society activists effective at making their voices heard or media organizations that amplify some discourses over others.
- **Network:** Network power is wielded when individuals use their personal relationships with others to shape their thinking and/or action [32]. Such relationships may be built on trust, reciprocity, repeated interaction over many years, shared experience, shared identities, or other factors. For example, when a board of directors appoints new members, the list of potential candidates may be drawn from existing board members’ personal networks; membership in other prestigious committees such as advisory groups, expert bodies, or commissions can follow a similar dynamic. Or, when a public conference is organized, decisions on which speakers to invite may be influenced by whether the candidates are personally known and trusted by the organizers. In this way, networks can translate into enhanced institutional, expert, or discursive power, but it is the breadth, structure, and content of the network that confers power on an individual or group [32]. Personal networks may also provide access to sensitive or strategic information, or immunity from public criticism. This conceptualization of network power is analogous to Bourdieu’s social capital, and overlaps to some extent with Weber’s concept of charismatic authority and what Sriram et al. have called “personal attributes” which are “tightly wound up with other individual factors such as gender, race, sexuality and religion” [1]

The typology in Table 1 separates different kinds of power that others have combined into single, broader categories, as shown in Table 2. It does so because different types of actors often have access to different types of power; therefore, a finer-toothed typology facilitates actor-based analysis. (For brevity, I include in Table 2 Sriram et al.’s characterization of various types of power rather than providing an exhaustive comparison of all significant theorists.)

**Table 2:** Relationship between Table 1 typology and others

Type of power	Dahl	Bourdieu	Barnett & Duvall	Sriram et al.
Physical	Coercion		Compulsory	
Economic		Economic	Compulsory	Financial
Structural			Compulsory, Structural	Political tradition-based; Political state-based
Institutional			Institutional	Bureaucratic
Moral		Symbolic	Compulsory, Productive	Personal attributes, charismatic
Expertise		Cultural	Productive	Technical expertise
Discursive			Compulsory, Productive	Personal attributes, charismatic
Network		Social		Networks and access, Personal attributes, charismatic

### Observations and implications

Several observations emerge from this typology. First, the typology underscores how a wide range of actors wield power in global governance, far beyond states, and often alongside them. The secretariat staff of intergovernmental organizations like WHO or the World Intellectual Property Organization often have considerable technical expertise and wide professional networks [33]. NGOs often wield the same types of power, in addition to moral and discursive [34, 35]. Well-resourced firms and foundations have economic power, often far in excess of that available to low-income states [36, 37]. And non-state actors increasingly have access to institutional power with the rise of multi-stakeholder partnerships that give them a formal governing role [38]. These are not new insights for students of global governance or global health; rather, these examples underscore the breadth of literature that has recognized the multiple forms of power at play in global governance processes. Included in this wide range of actors are those that IR studies may consider weak, such as governments of the lowest-income countries, social movements, individual moral leaders, NGOs, and technical experts.

Second, these categories may be deeply interconnected—they are not mutually exclusive. For example, the exercise of one type of power can be *conditional* upon another. To illustrate: those with the discursive power to frame or problematize an issue also influence the “solutions” that NGOs may advance (using expert and moral power) or that policy-makers may adopt (through institutional power) to address the “problem” [39]. The kind of expert knowledge that is considered authoritative in debates over such “solutions” is likely to reflect underlying distributions of economic, discursive, and network power [30, 40]. The extent to which an individual has network power is likely to be conditional upon his/her discursive, economic or expert power.

Furthermore, as highlighted in Bourdieu’s concept of power as capital, one type of power can be fungible—that is, can be *transformed* into another. For example, economic power can be transformed into institutional power when donor money sways the negotiation of decision-making rules to give a donor a voting seat on an organization’s governing board. It can be transformed into expert power when funders influence which research is or is not conducted and/or published [41]. Moral power can be transformed into economic power when a religious leader mobilizes donations for a cause. Expert power can be transformed into physical power, for example, when a group of weapons and health experts convinces political leaders to take military action to counteract the suspected use of chemical weapons. The understanding that types of power are fungible highlights not only how power disparities can be consolidated when multiple types of power reinforce each other, but also that the distribution of power can be changed.

Furthermore, actors wield different types of power as they pursue their goals and protect their interests in the global arena. The combination of many actors simultaneously wielding different types of power in a complex adaptive system means that outcomes are difficult to predict or control, causality is challenging to trace or establish, and power can be difficult to discern or analyze [26]. At the same time, the very fact of complexity may also create opportunities for less powerful actors to wield influence [42].

Thus, the third observation is that the typology highlights how actors often perceived as weak or powerless may be able to wield influence in ways not widely recognized [43]. For example, as scholars of transnational civil society networks have shown [34, 44], NGOs with far fewer economic resources than multinational corporations or wealthy governments are able to use moral, expert, and discursive power to act as an effective counterweight to them in global political arenas. One concrete example is the successful network of civil society organizations that campaigned for taking flexible approaches to global intellectual property rules on medicines patents in developing countries; at first, this effort was strongly opposed by the pharmaceutical industry and Northern governments, but it eventually succeeded in removing patents as a major barrier to widespread access to generic HIV medicines in LMICs [45]. The point here is not to minimize recognition of the power asymmetries permeating global health, which can be enormous and enormously consequential. Rather, it is intended as an argument against over-simplification, such as dividing actors into binary categories of strong and weak. It is also an argument against the inadvertent disempowerment of actors by describing them as “weak”—an actor can arguably wield more power when considered by others to be powerful than the converse. Fuller recognition of the many different types of power operating in global governance can provide more nuanced and convincing explanations of outcomes than narrow conceptions of power alone—especially when “weaker” actors succeed in challenging “stronger” ones.

## Conclusions and directions for future research

Understanding power has increasingly come to be recognized as essential to understanding global governance and health. The typology offered here seeks to help analysts and practitioners identify the different types of power at work in global governance, the actors who wield it, and the ways in which one type can be transformed into or amplify others. Further research is needed to shed light on the distribution of different kinds of power across actors, how redistribution may be achieved, and how different forms of power interact to produce outcomes. Also needed are methods for studying power that take into account complexity without being paralyzed by it.

Finally, one seemingly contradictory – and cautiously optimistic – conclusion emerges from this typology: multiple types of power can mutually reinforce tremendous power disparities in global health; but at the same time, such disparities are not necessarily absolute or immutable.

## Data Availability

Not applicable.

## Abbreviations

Global Fund: Global Fund to Fight AIDS, Tuberculosis and Malaria
HIV: Human immunodeficiency virus
IR: International relations
LMICs: Low and middle income countries
NGO: Non-governmental organization
UN: United Nations
WHO: World Health Organization

## References

[1] Sriram V, Topp SM, Schaff M (2018). 10 best resources on power in health policy and systems in low- and middle-income countries. Health Policy Plan.

[2] Commission on Social Determinants of Health. Closing the gap in a generation: health equity through action on the social determinants of health. Geneva: World Health Organization; 2008. 10.1016/S0140-6736(08)61690-6 18994664

[3] Ottersen OP, Dasgupta J, Blouin C (2014). The political origins of health inequity: prospects for change. Lancet.

[4] Marten R, Hanefeld J, Smith R (2014). Commission on global governance and health: what about power?. Lancet.

[5] Shiffman J (2014). Knowledge, moral claims and the exercise of power in global health. Int J Health Policy Manag.

[6] Gore R, Parker R (2019). Analysing power and politics in health policies and systems. Glob Public Health.

[7] Lee K (2015). Revealing power in truth: comment on “knowledge, moral claims and the exercise of power in global health”. Int J Health Policy Manag.

[8] Frenk J, Moon S (2013). Governance challenges in global health. N Engl J Med.

[9] Lee K, Kamradt-Scott A (2014). The multiple meanings of global health governance: a call for conceptual clarity. Glob Health.

[10] Dahl RA. Who governs? Democracy and Power in an American City. New Haven, CT: Yale University Press; 2005 (orig. edition 1961).

[11] Barnett M, Duvall R (2005). Power in international politics. Int Organ.

[12] Bourdieu P. The forms of capital. In: Granovetter M, Swedberg G, eds. The Sociology of Economic Life. Westview Press; 1992.

[13] Foucault M (1979). The history of sexuality, part 1.

[14] Lukes S. Power: a radical view. Macmillan International Higher Education. 2004.

[15] Weber M. “Politics as Vocation.” In Owen D, Strong T (translated by Livingstone R). The Vocation Lectures. Illinois: Hackett Books. 2004.

[16] Adler-Nissen R (2012). Bourdieu in international relations: rethinking key concepts in IR.

[17] Leander A (2011). The promises, problems, and potentials of a Bourdieu-inspired staging of international relations. Int Polit Sociol.

[18] Guzzini S. Power. In: Adler-Nissen, ed, Bourdieu in international relations. Abingdon: Routledge; 2012. p. 78–92.

[19] Hanefeld J, Walt G (2015). Knowledge and networks—key sources of power in global health: comment on “knowledge, moral claims and the exercise of power in global health”. Int J Health Policy Manag.

[20] Bourdieu P (2013). Distinction: a social critique of the Judgement of taste.

[21] Keohane RO, Martin LL (1995). The promise of Institutionalist theory. Int Secur.

[22] Mearsheimer J (1995). The false promise of international institutions. Int Secur.

[23] Barnett M, Duvall R, Barnett M, Duvall R (2005). Power in global governance. Power in global governance.

[24] Wallerstein I (1974). The modern world- system: capitalist agriculture and the origins of the European world-economy in the sixteenth century.

[25] Jervis R (1997). System effects: complexity in political and social life.

[26] Hill PS (2011). Understanding global health governance as a complex adaptive system. Glob Public Health.

[27] de Savigny D, Adam T (2009). Systems thinking for health systems strengthening.

[28] Tarrow SG (2011). Power in movement: social movements and continuous politics.

[29] Adler E, Haas PM (1992). Conclusion: epistemic communities, world order, and the creation of a reflective research program. Int Organ.

[30] Sending OJ (2015). The politics of expertise: competing for Authority in Global Governance.

[31] Tichenor M, Sridhar D (2019). Metric partnerships: global burden of disease estimates within the World Bank, the World Health Organisation and the Institute for Health metrics and evaluation. Wellcome Open Res.

[32] Granovetter MS. The strength of weak ties. In: S. Leinhardt, ed. Social Networks. Elsevier; 1977. pp. 347–367.

[33] Barnett M, Finnemore M (2004). Rules for the world: international organizations in global politics.

[34] Keck ME, Sikkink K (1998). Activists beyond Borders.

[35] Finnemore M, Sikkink K (1998). International norm dynamics and political change. Int Organ.

[36] Hall RB, Biersteker TJ (2003). The emergence of private Authority in Global Governance.

[37] Braithwaite J, Drahos P (2000). Global business regulation.

[38] Andonova LB (2017). Governance entrepreneurs: international organizations and the rise of global public–private partnerships.

[39] Bardach E. A Practical Guide for Policy Analysis: The Eightfold Path to More Effective Problem Solving. 4^th^ edn. Los Angeles, CA; Sage/CQ Press, 2012.

[40] Jasanoff S (2004). States of knowledge: the co-production of science and the social order.

[41] Storeng KT, Abimbola S, Balabanova D (2019). Action to protect the independence and integrity of global health research. BMJ Glob Health.

[42] Alter KJ, Meunier S. The politics of international regime complexity. Perspect Polit 2009; 7: 13.

[43] Scott J (1985). Weapons of the weak: everyday forms of peasant resistance.

[44] Price RM (2003). Transnational civil society and advocacy in world politics. World Polit.

[45] Hein W, Moon S (2016). Information norms in global governance: human rights, intellectual property rules and access to medicines.

